# Green Ca-source of cockle shells converted to calcium acetate for environmental sustainability

**DOI:** 10.1016/j.heliyon.2024.e32153

**Published:** 2024-05-31

**Authors:** Somkiat Seesanong, Chaowared Seangarun, Banjong Boonchom, Natee Ohpasee, Nongnuch Laohavisuti, Wimonmat Boonmee, Pesak Rungrojchaipon

**Affiliations:** a Office of Administrative Interdisciplinary Program on Agricultural Technology, School of Agricultural Technology, King Mongkut's Institute of Technology Ladkrabang, Bangkok, 10520, Thailand; b Material Science for Environmental Sustainability Research Unit, School of Science, King Mongkut's Institute of Technology Ladkrabang, Bangkok, 10520, Thailand; c Municipal Waste and Wastewater Management Learning Center, School of Science, King Mongkut's Institute of Technology Ladkrabang, Bangkok, 10520, Thailand; d Department of Chemistry, School of Science, King Mongkut's Institute of Technology Ladkrabang, Bangkok, 10520, Thailand; e Department of Animal Production Technology and Fishery, School of Agricultural Technology, King Mongkut's Institute of Technology Ladkrabang, Bangkok, 10520, Thailand; f Department of Biology, School of Science, King Mongkut's Institute of Technology Ladkrabang, Bangkok, 10520, Thailand

**Keywords:** Cockle shell waste, Calcium carbonate, Calcium acetate monohydrate, Greenhouse gases emission, Renewable calcium source

## Abstract

This work aimed to synthesize and characterize the calcium acetate monohydrate (Ca(CH_3_COO)_2_·H_2_O) from the exothermic reaction between CaCO_3_ powder derived from cockle shells with three different acetic acids (8, 10, and 12 mol L^−1^) concentrations by the rapid and easy process without pH and temperature control to lead to cheap chemical production. The physicochemical characteristics of all synthesized Ca(CH_3_COO)_2_·H_2_O samples are investigated based on the chemical compositions, crystal structures, vibrational characteristics, morphologies, and thermal behavior to confirm the target compound. A suitable concentration of 10 mol L^−1^ CH_3_COOH was chosen to produce Ca(CH_3_COO)_2_·H_2_O with the highest yield (96.30 %), maximum calcium content (96.2 % CaO) with lower impurities, and time consumption of 17 h. The calcium acetate product obtained from cockle shells in this work shows differences in thermal stability, morphological structure purity, %yield, and metal contamination with those reported obtained from other sources and another shell type in the previous work. This research investigates the transformation of cockle shell waste into CaCO_3_ for the production of calcium acetate, aiming to address environmental sustainability concerns by reducing the use of calcium ore resources and greenhouse gas emissions.

## Highlights

• Calcium acetate monohydrate (Ca(CH_3_COO)_2_.H_2_O) was rapidly synthesized from cockle shell waste at a low cost.
• According to upcycling, renewable calcium sources from cockle shell waste were used to replace calcium ore resources.
• Calcium acetate production's environmentally friendly process shows low greenhouse gas emissions.
• Calcium acetate shows high potential in specific applications (medical, fertilizer, food additive, environmental, etc).

## 1 Introduction

The recycling processes of many wastes are increasing dramatically to sustain the social economy and protect the environment [1], which affects sustainable development. The scarcity of various natural resources has worried both developed and developing countries, resulting in the increasing of environmental techniques to reuse and recycle waste. Consequently, many researchers have tried to find green technologies with different methods for recycling and making waste useful again [2]. The mollusks from aquaculture and fisheries generated large volumes of biowastes [3], which reported a global mollusk production of about 6 million tons [4]. The edible part is evaluated at about 20 %, thus generating shell waste of about 80 % of the total. The lack of green and suitable technologies to recover and treat wastes causes various environmental and health problems [3]. Unsuitable disposal of highly toxic wastes also affects negatively the soil, air, and water. Furthermore, some wastes act as important substrates for microbial proliferation, which is one of the biggest pollutants in hydrological basins [4]. On the other hand, waste also exhibits the biggest benefit by employing them as renewable sources for the preparation of value-added materials, which promotes numerous benefits for both economic and environmental topics [5]. Many mollusks’ wastes are important sources for the production of carotenoids (i.e., astaxanthin) [6], minerals (i.e., calcium carbonate, CaCO_3_) [7], fatty acids [8], chitin and its derivatives [7], and essential amino acids [5], etc. The recovery of waste has been gaining prominence by applying them in the production chain [3]. Among the recovery processes, the use of chemical reagents, i.e., enzymes [9] and acids [10], is the most common technique that is widely used for waste recovery. After recovery processes, obtained compounds (recovered from the wastes) can be applied in salinity separations [11], nutricosmetics [12], therapies [13], nutraceuticals [14], drugs [15], animal feed [16], and fertilizers [17].

**Fig. 1:**
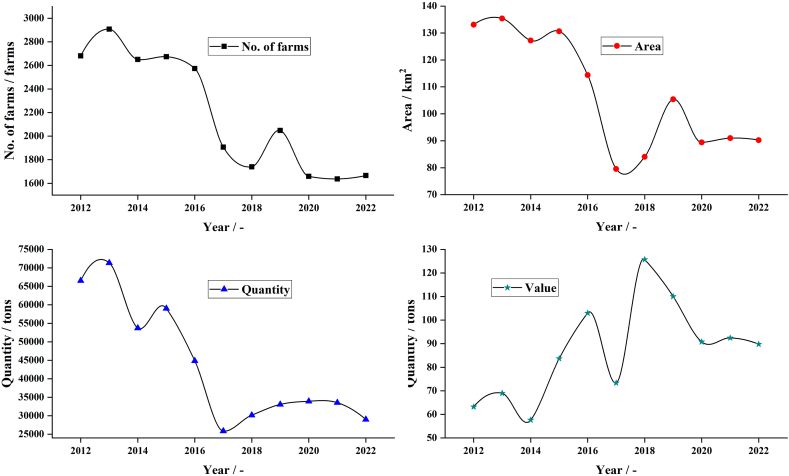
Number (No.) of farms, farm area, quantity, and value of cockle shell production in Thailand during 2010–2019.

Cockle (*Anadara granosa*), one of the valuable seafood [18], is a marine bivalve that belongs to the Arcidae family [19,20]. It is an important supplement source for humans and a food source for birds, fish, crabs, and shrimp [21]. It has been applied as a bioindicator for marine pollutants, i.e., polycyclic aromatic hydrocarbons (PAHs) [22] and heavy metals [23]. Cockles are available in various coastal regions of Southeast Asia, especially Thailand [19]. Based on the Statistics of Marine Shellfish Culture Survey 2022, Department of Fisheries Ministry of Agriculture and Cooperatives (Thailand), the value of the cockle production in 2022 was 617.34 million USD with 1666 farms, a total farm area of 105.39 km^2^, and 30,364 tons of cockle production. Fig. 1 shows the number of farms, farm area, quantity, and value of cockle production in Thailand during 2010–2022. The large quantity of cockle production each year makes up about 80 % of shell waste, which causes various environmental problems [24]. Cockle shells consist of about 96–98 % calcium carbonate (CaCO_3_) with other chemical elements in compositions, i.e., silicon (S), zinc (Zn), boron (B), nickel (Ni), copper (Cu), iron (Fe), potassium (K), phosphorus (P), sodium (Na), and magnesium (Mg) [25]. The cockle shell CaCO_3_ in an aragonite polymorph [26] was found to be highly purified and safe for medical application [27]. Calcium carbonate compound has three crystal phases namely calcite, aragonite, and vaterite with different morphological structures. The source, type, and period of calcium carbonate formation affect phase formation in terms of phase type and mixed-phase content and affect the level of purity and metal contamination [25]. The manufacturing process for calcium carbonate powder from cockle shells is like that obtained from limestone and other shellfish. But this shellfish waste occurs every day and never runs out, like limestone. Therefore, cheap cockle shell wastes are readily available and abundantly considered an attractive alternative material for use as a renewable source for transforming into other valuable compounds [24,28].

Calcium acetate is an important calcium compound, which can be used in various applications. It was used in the medical field to treat hyperphosphatemia (too much phosphate in the blood) symptoms [29]. It was used in an environmental field as a powerful agent to control the emission of sulfur dioxide (SO_2_), nitrogen oxide (NO_X_), and other toxic gasses in coal combustion processes, reduce the acid rain phenomena [30], and replace the corrosive and environmentally unacceptable deicers [31]. It was used in food industries as a stabilizer and preservative in many food substances under the number E263 [32]. It was used in the agricultural field as a soil conditioner, a plant micronutrient, a foliar fertilizer, and a soil pH adjuster [33]. Furthermore, it was used in the chemical industry as a starting reactant for the production of valuable compounds, i.e., as nano calcium oxide [34] and calcium carbonate [35] and calcium phosphates [36], acetone [37], composite ceramic compounds [38], anhydrite calcium sulfate nanowhiskers [39], and cement mortar additive [40]. According to the previous work, calcium acetate was synthesized from the reaction between various calcium sources and acetic acid (CH_3_COOH) [[41], [42], [43]]. Based on chemical and material science, a chemical product prepared using a type of raw material that differs (shells and limestone) will affect certain physical properties (thermal stability morphological structure solubility purity % yield, and toxic metal contamination) [44].

In addition, many researchers have tried to find a new calcium source to reduce the cost of calcium acetate production, and cockle shell wastes are great selectness for use as renewable calcium sources to produce the target compound. Consequently, the objective of this work is to synthesize and characterize calcium acetate by employing cockle shell waste as a renewable source and investigate the influence of the concentration of acetic acid on its physicochemical properties. This work reports on an alternative route to producing calcium acetate instead of using nonrenewable resources and helps to reduce greenhouse gas emissions, which avoids various significant adverse environmental effects affecting sustainable management. Additionally, this research will be useful for communities where have cockle shell sources and then use them as the only raw material for calcium acetate production.

## 2 Materials and methods

### 2.1 Materials and reagents

The chemical replacement reaction of calcium acetate preparation used starting reactants such as cockle shell powder and acetic acid. The cockle shells were collected from the shells stacking site in Ang Sila Tambon, Chonburi district, Chonburi Province, Thailand. This research supports reducing the use of calcium carbonate from limited mineral resources and helps eliminate waste shellfish to create added value for the area that has only one type of raw material of this kind. Firstly, the cockle shells were cleaned to remove mollusks’ residues, using 2.5 % sodium hypochlorite (NaOCl) solution and then washed with distilled water to remove other residual particles. The cleaned cockle shells were dried over sunlight for about 3 h. Next, the dried cockle shells were ground and sieved through a 100-mesh to get fine CaCO_3_ powders with particle sizes smaller than 0.149 mm, used as a raw material for calcium acetate synthesis, which was labeled as CS-CaCO_3_. The CS-CaCO_3_ raw agent was checked for chemical composition and crystal phase by XRF and XRD techniques, respectively. The result of the chemical composition of the CS-CaCO_3_ raw agent was found to be 94.7%CaO, 1.47%Na_2_O, 1.15%SiO_2_, 1.06%P_2_O_5_, 0.584%MgO, 0.550%Al_2_O_3_, 0.260%CuO and 0.127%K_2_O, so the purity of this agent is about 94.7 %. The crystal amorphous investigated by XRD techniques confirms that the aragonite phase is the main crystal structure.

### 2.2 Calcium acetate synthesis

This work focuses on acetic acid concentrations in the range 8–12 mol L^−1^ based on the results of the physical and chemical properties of calcium acetate obtained from other shells, reported in previous work [43,45] as a guideline. To prepare the target calcium acetate compound according to Eq. (1), the volumes of each acetic acid concentration were measured at 2.5, 2.0, and 1.67 L for 8, 10, and 12 mol L^−1^, respectively. Each acetic acid concentration was slowly added into each beaker (5L) that contained 1.056 kg of CS-CaCO_3_ powders. The obtained mixture was stirred continuously with the elimination of CO_2_ gas, and the highest reaction temperature during the reaction was detected. The stirring process was finished when CO_2_ had completely evaporated. After that, the resulting mixture was exposed to air at room temperature and then dried by itself. The duration time depending on the concentration of acetic acid in the calcium acetate synthesis to obtain the dried product was recorded. The dried products were then ground, and the pale-grey calcium acetate monohydrate (CAM) powders were obtained. The powder products synthesized from reactions (Eq. (2)) that used 8, 10, and 12 mol L^−1^ CH_3_COOH had the percentage yields calculated and were labeled as CAM8, CAM10, and CAM12, respectively.

(1) CaCO_3_(s) + 2CH_3_COOH(aq) → Ca(CH_3_COO)_2_·H_2_O(s) + CO_2_(g)

In chemistry, percentage yield (%) is a measure of the quantity of a product formed from the starting material [46]. Percentage yield is an important parameter that exhibited the efficiency of the preparation method used in this work. Consequently, a suitable condition for Ca(CH_3_COO)_2_·H_2_O production should present a high production yield. In this research, the percentage yield of the chemical synthesis of Ca(CH_3_COO)_2_·H_2_O according to Eq. (1) was calculated from the ratio between the mass of the obtained reaction product (Ca(CH_3_COO)_2_·H_2_O) and mass of the starting material (CS–CaCO_3_ powder) as shown in Eq. (2).

(2) yield = m_p_/m_sm_ x 100%

where *m*_p_ and *m*_sm_ are masses of Ca(CH_3_COO)_2_·H_2_O product and CS-CaCO_3_ (starting material), respectively.

### 2.3 The estimation of the CO_2_ emission

Calculating CO_2_ emissions from the production of the bio-green CaCO_3_ powder obtained from cockle shell wastes is based on the following equations [47,48]:

(3) E _bio-green CaCO3_ = E_T_ + E_W_ + E_E_

(4) E_T_ = distance x loading x EF_tuck_

(5) E_W_ = amount of water usage x EF_water_

(6) E_E_ = electricity consumption x EF_E_

E _bio-green CaCO3_ is the carbon emission of the production of the bio-green CaCO_3_ powder, kgCO_2_

E_T_ is the carbon emission of transportation of the production of the bio-green CaCO_3_ powder, kgCO_2_

E_W_ is the carbon emission of water usage for the production of the bio-green CaCO_3_ powder, kgCO_2_

E_E_ is the carbon emission of electricity consumption for the production of the bio-green CaCO_3_ powder, kgCO_2_

EF_tuck_ is an emission factor for a kind of tuck; EF_W_ is an emission factor for water; EF_E_ is an emission factor for electricity.

Commercial acetic acid (CH_3_COOH, industrial grade, Merck) with the beginning concentration of 17.416 mol L^−1^ (99.7 wt%, d = 1.05 kg/L) [47] was used without further purification as one of the reagents to prepare the calcium acetate. Before use, the acetic acid was diluted by deionized (DI) water, and three different acetic acid concentrations of 8, 10, and 12 mol L^−1^ were then achieved. The CO_2_ emissions of three different acetic acid concentrations were estimated by Eq. 7

(7) CO_2_ emission of acetic dilution = (V_a_ x EF_CH3COOH_ + V_w_ x EF_H2O_)

So, V_a_ and V_w_ are the volume of acetic acid and DI water, respectively. EF_CH3COOH_ (2.4478 kgCO_2_ e/L or 2.5702 kgCO_2_ e/kg) and EF_H2O_ (2.1555 kgCO_2_ e/m^3^ or 2.1555 x 10^−3^ kgCO_2_ e/kg) [47,48] are CO_2_ emission factor of acetic acid and DI water, respectively. From Eq. (2) the CO_2_ emission of acetic acid solution of 8, 10, and 12 mol L^−1^ are about 1.2407, 1.5504, and 1.8601 kgCO_2_ e/L, respectively.

The CO_2_ emission of the production of Ca(CH_3_COO)_2_·H_2_O was estimated by consideration with only chemical agents in the reaction (Eq. (1)) and the calculation equation is shown in Eq. (8)

(8) CO_2_ emission of the Ca(CH_3_COO)_2_·H_2_O production = [m_CaCO3_ x EF_CaCO3_ + m_acid_ x EF_acid_ + m_CO2_]/m_ca_

Where m_CaCO3_, m_acid,_ m_CO2_ and m_ca_ are the quantity of used starting agents (CaCO_3_ and acid concentrations, CH_3_COOH) and formed products (calcium acetate and CO_2_ gas), respectively. EF_CaCO3_ and EF_acid_ are CO_2_ emission factors of calcium carbonate and acetic acid, respectively.

### 2.4 Samples characterization

The chemical compositions of all synthesized calcium acetate products were analyzed by SRS 3400 X-ray fluorescence (XRF) spectrophotometer (Rigaku). To prepare the XRF sample, each calcium acetate was pulverized, homogenized, and pressed into a pellet [49]. A starch was applied as a binder, and 15 g of the sample was mixed with 150 g of binder using agate mortar to prevent contamination. A 150 mg pellet is prepared with around 25 mm diameter. The crystal structures of calcium acetates were investigated by MiniFlex X-ray diffraction (XRD) instrument (Rigaku) with 600 W X-ray tube and ultra-silicon strip detector. The structural investigation was conducted at room temperature with 2 theta (2Ɵ) range of 5–60°. A narrow increment of 0.02° and the scan speed of 1 s·step^−1^ were selected to obtain reliable experimental data [50].

Infrared absorption spectra of calcium acetate samples were recorded on the Spectrum GX Fourier transform infrared (FTIR) spectrophotometer (PerkinElmer) from 4000 to 400 cm^−1^ using a resolution of 2 cm^−1^. For sample preparation, about 1 g of the sample was mixed homogeneously with 10 g of spectroscopic-grade potassium bromide (KBr). The mixture was pressed as a pellet using a manual hydraulic press (2 tons, 1 min). The infrared absorption spectrum of each pellet was recorded. The morphologies of calcium acetate products were analyzed by 1450 VP scanning electron microscope (SEM, LEO). Each product was placed on the traditional alumina stubs first using double-sided conductive tape and was coated with gold powder using a sputtering technique before SEM analysis [51]. Finally, the thermogravimetric analysis (TGA) was employed to investigate the thermal behavior of calcium acetates by using the Pyris Diamond thermogravimetric/differential thermal analysis (TG/DTA) instrument (PerkinElmer). Firstly, alpha-alumina (*α*-Al_2_O_3_) was calcined at 800 °C for 3 h under nitrogen (N_2_) gas, and the calcined *α*-Al_2_O_3_ was used as reference material. About 8 mg of each sample was placed into the sample TG pan, whereas the *α*-Al_2_O_3_ was placed in the reference TG pan. Conditions performed at a heating rate of 10 °C·min^−1^ from room temperature up to 900 °C under N_2_ atmosphere with a flow rate of 100 mL min^−1^

## 3 Results and discussion

### 3.1 Preparation results

Eq. (1) shows the chemical reaction between the CS-CaCO_3_ powder and acetic acid (CH_3_COOH) solution, resulting in the formation of calcium acetate monohydrate (CAM, Ca(CH_3_COO)_2_·H_2_O) product. After using three different acetic acid concentrations (8, 10, and 12 mol L^−1^), three reaction products, namely CAM8, CAM10, and CAM12, were obtained, respectively. The reaction-yield percentages of CAM8, CAM10, and CAM12 products calculated from Eq. (4) are listed in Table 1. Other experimental parameters, namely reaction temperature and drying time, observed during the Ca(CH_3_COO)_2_·H_2_O preparation process, are also shown in Table 1.

**Table 1:** Corresponding reaction temperature, drying time, and synthesis yield during the synthesis of Ca(CH_3_COO)_2_·H_2_O from cockle-shell CaCO_3_ waste and three different acetic acid concentrations.

Reaction products	Acetic acid concentration/mol·L^−1^	Reaction temperature/°C	Drying time/min	Synthesis yield/%
CAM8	8	38	24	91.88
CAM10	10	44	17	96.30
CAM12	12	53	13	94.90

The results demonstrated that the reaction temperature during the preparation reaction increased with increasing acetic acid concentration. In contrast, the drying time (time to obtain the dried Ca(CH_3_COO)_2_·H_2_O powder) decreased with increasing acetic acid concentration. The increase in the reaction temperature causes the rapid evaporation process of the reaction medium or water, resulting in a decrease in drying time with the increasing acetic acid concentration. As shown in Table 1, the highest percentage yield (96.30 %) was observed for the CAM10 product, which was obtained from the reaction between 10 mol L^−1^ acetic acid and CS-CaCO_3_ powder. On the other hand, the lowest percentage yield (91.88 %) was observed from CAM8 due to the highest amount of reaction medium (water), which might cause the low amount of the starting reagent (acetic acid) for Ca(CH_3_COO)_2_·H_2_O production. The drying time of the Ca(CH_3_COO)_2_·H_2_O from cockle shells that mainly contain CaCO_3_ in aragonite form in this work is lower than the preparation of Ca(CH_3_COO)_2_·H_2_O using oyster shells and scallop shells that mainly contain CaCO_3_ in calcite form in the previous work [43,45]. However, all percentage yields observed in this work (cockle shells as raw agent) are higher than those reported in the previous work (89–93 % yield) [43,45] which results in cheaper production costs. The results obtained are consistent with previous research, showing that using cockle shells (aragonite) (91–95 %) instead of oyster shells (89–93 %) or scallop shells (calcite) (85–87 %) leads to increased percentage yields during the preparation of calcium acetate. Additionally, the use of cockle shells also reduces the drying time necessary for the process [52]. It would be indicated that the source and type of calcium affect the percentage yield and the drying time in the Ca(CH_3_COO)_2_·H_2_O production.

### 3.2 The estimation of the CO_2_ emission results

The CO_2_ emission of the prepared CS-CaCO_3_ raw agent was estimated by Eqs. (3)–(6). The CO_2_ emission value of the preparing CS-CaCO_3_ in this work is smaller than that of CaCO_3_ produced from limestone (1.0676 kg CO_2_ e/kg, at the plant) [47] (supporting information). The CO_2_ emission for the Ca(CH_3_COO)_2_·H_2_O production estimated by Eq. (8) considered only chemical substances according to Eq. (1). For the CS-CaCO_3_ powder usage, The CO_2_ emission of the Ca(CH_3_COO)_2_·H_2_O production are calculated and found to be 2.2840, 2.1888, and 2.2183 kg CO_2_ e/kg for different acetic acid concentrations; 8, 10, and 12 mol L^−1^, respectively (supporting information). If calcium carbonate obtained from limestone was used and considered 100 % purity and 100 % yield, the CO_2_ emission value of the Ca(CH_3_COO)_2_·H_2_O production is equal to about 2.62 kg CO_2_ e/kg (supporting information). Under this estimation, the transformation of cockle shell waste to calcium acetate can help in reducing CO_2_ emission (0.4–0.5 kg CO_2_ e/kg) and reducing calcium ores usage (limited resources and non-renewable), which contributes to the advancement of environmental sustainability.

### 3.3 X-ray fluorescence (XRF) results

The elemental compositions of CAM8, CAM10, and CAM12 products were determined and the corresponding results are presented in Table 2. The XRF results show that CAM8, CAM10, and CAM12 mostly consisted of CaO of around 95.1, 96.2, and 94.6 wt%, respectively. This data confirms the % purity of the synthesized Ca(CH_3_COO)_2_·H_2_O samples, which are lower than those of Ca(CH_3_COO)_2_·H_2_O (95–98 % purity) produced by scallop shells and oyster shells with acetic acid reported in the previous work [43,45]. The reason could be explained by the %purity of calcium carbonate from the difference in sources and kinds (cockle and scallop shells). In addition, other trace chemical compositions as shown in Table 2 were also reported in oxide form after calculation from XRF data, i.e., disodium oxide (Na_2_O, 0.9−1.7 wt%), silicon dioxide (SiO_2_, 0.8−1.2 wt%), ferric oxide (Fe_2_O_3_, 0.7−1.0 wt%), etc.

**Table 2:** Chemical compositions of cockle-shell derived Ca(CH_3_COO)_2_·H_2_O products prepared from three different acetic acid concentrations.

Compounds	Formula	Chemical contents/wt%		
		CAM8	CAM10	CAM12
Calcium oxide	CaO	95.1	96.2	94.6
Disodium oxide	Na_2_O	1.41	0.932	1.69
Silicon dioxide	SiO_2_	1.06	0.862	1.16
Ferric oxide	Fe_2_O_3_	0.926	0.777	1.02
Aluminium oxide	Al_2_O_3_	0.431	0.342	0.465
Strontium oxide	SrO	0.404	0.391	0.403
Magnesium oxide	MgO	0.190	0.138	0.184
Sulfur oxide	SO_3_	0.170	0.110	0.177
Titanium dioxide	TiO_2_	0.109	0.109	0.141
Manganese oxide	MnO	0.0722	0.0451	0.0569
Phosphorous oxide	P_2_O_5_	0.0489	0.0403	0.0495
Dipotassium oxide	K_2_O	0.0217	0.0179	0.0223
Chlorine	Cl	0.0205	0.0125	0.0184
**Summation**		**99.96**	**99.98**	**99.99**

According to the results, when the acetic acid with the concentration of 10 mol L^−1^ was used in the preparation process, the resulting Ca(CH_3_COO)_2_·H_2_O product (CAM10) showed the highest CaO content with the lowest amounts of other chemical compositions. On the other hand, the lowest CaO content was observed, when the acetic acid with the concentration of 12 mol L^−1^ was used in the Ca(CH_3_COO)_2_·H_2_O preparation process (CAM12). From these obtained results, it can be concluded that some specific concentrations of acetic acid can leach more oxide impurities, and the suitable concentration of acetic acid was observed to be 10 mol L^−1^. Similar results were reported by Seesanong et al. [53] in the preparation of triple superphosphate (TSP, Ca(H_2_PO_4_)_2_·H_2_O) compound synthesized from the exothermic reactions between oyster-shell derived CaCO_3_ powder and phosphoric acid (H_3_PO_4_). The influence of the H_3_PO_4_ concentrations (10−70 wt%, increment: 10 wt%) on the chemical composition of Ca(H_2_PO_4_)_2_·H_2_O products was also investigated. The researchers observed that the contents of the major compositions (P_2_O_5_ and CaO) and other impurities (SO_3_, K_2_O, Fe_2_O_3_, Al_2_O_3_, and SiO_2_) of all Ca(H_2_PO_4_)_2_·H_2_O products depended on the H_3_PO_4_ concentrations using in the preparation process, and 60 wt% was the suitable H_3_PO_4_ concentration, exhibiting the Ca(H_2_PO_4_)_2_·H_2_O product with the lowest chemical impurities [46]. Based on the composition results, toxic elements were not detected, which supported the using Ca(CH_3_COO)_2_·H_2_O in specific applications for the future.

### 3.4 X-ray diffraction (XRD) results

**Fig. 2:**
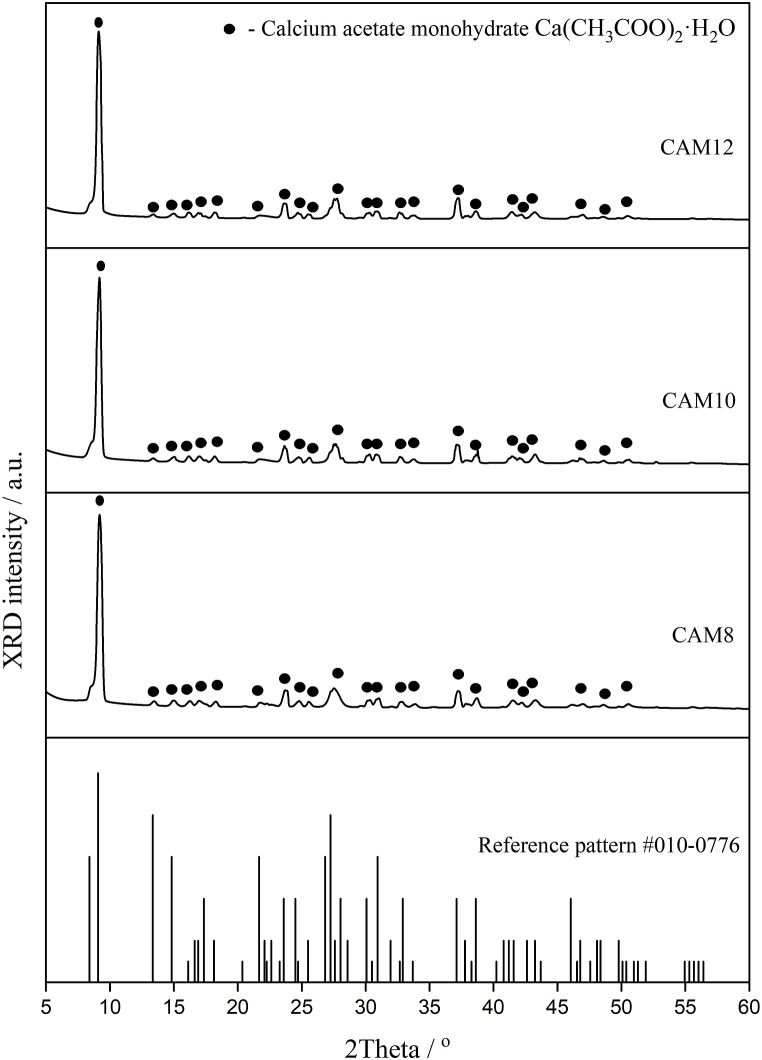
X-ray diffraction (XRD) patterns of the synthesized calcium acetate monohydrate products ((a) CAM8, (b) CAM10, and (c) CAM12) prepared from three different acetic acid concentrations.

The corresponding XRD patterns of (a) CAM8, (b) CAM10, and (c) CAM12 samples derived from cockle-shell waste are displayed in Fig. 2. Obtained diffraction patterns of synthesized products were compared against known diffraction patterns of calcium acetate standard.

As demonstrated in Fig. 2, all prepared samples show similar XRD patterns, and all diffraction patterns were closely matched to the calcium acetate monohydrate (Ca(CH_3_COO)_2_·H_2_O), after comparing to the JCPDS data (PDF # 010–0776) [54]. Diffraction patterns of all Ca(CH_3_COO)_2_·H_2_O products achieved in this work agree well with those reported by Musumeci et al. [54] but differ from those reported by Thongkam et al. [43]. This indicates that the crystal structure of calcium acetate depends on the difference of synthetic methodologies and used raw materials, which may be consistent with some previous studies [43,54,55]. Klop et al. [55] investigated the crystallography of needle-shaped Ca(CH_3_COO)_2_·H_2_O sample (0.08 × 0.13 × 1.13 mm^3^) by Patterson and Fourier techniques, and the crystal structure of the sample was well explained. They also investigated the density value (*D*) of Ca(CH_3_COO)_2_·H_2_O, and its *D* value of 1.506 g cm^−3^ was reported [55]. Ca(CH_3_COO)_2_·H_2_O had the space group of *P1* (space group # 1) with the Schoenflies symbol of C_1_^1^. It crystallizes in a triclinic crystal system with the number of molecules (or formula units) in the unit cell (*Z*) of 4. Its structure consists of infinite multiple O-bridged double-stranded calcium chains, while the H bonds acted as the cross-linker between calcium chains. The lattice parameters *a*, *b*, and *c* are 6.751, 11.077, and 11.783 Å, whereas the lattice angles *α*, *β* and, *γ* are 116.50, 92.41, and 97.32°, respectively. The corresponding unit cell volume was 777.1 Å^3^ [55]. The crystalline sizes of CAM8, CAM10, and CAM12 samples based on the XRD patterns of Ca(CH_3_COO)_2_·H_2_O calculated by the Scherrer's equation (($S_{c}=\frac{0.9\lambda }{\beta \cdot \cos\;\theta }$), where λ is the employed X-ray wavelength (0.154059 nm), β is the full width at the half maximum (FWHM in radians) of the investigated diffraction peak, and θ is diffraction peak angle (in radians)) were found to be 18.92, 19.35, and 20.35 nm, respectively. This XRD result indicates that the increased acetic acid concentration will cause the increased crystal size of the calcium acetate.

### 3.5 Fourier transform infrared (FTIR) results

**Fig. 3:**
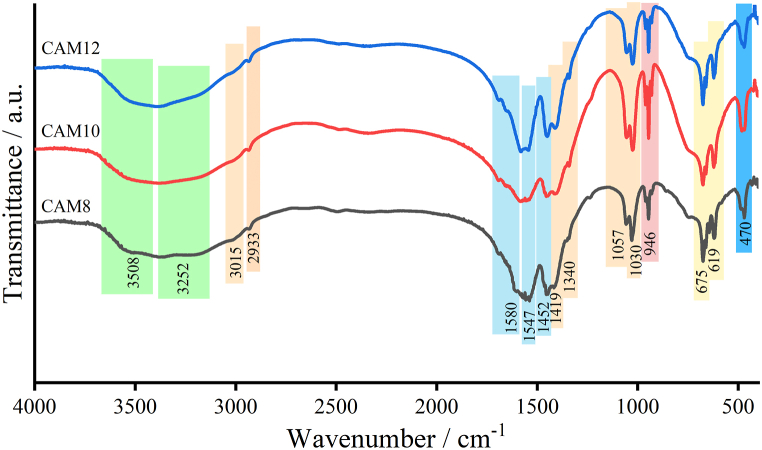
Fourier transform infrared (FTIR) spectra of the synthesized calcium acetate monohydrate products prepared from three different acetic acid concentrations(CAM8, CAM10, and CAM12).

The infrared spectroscopic results of Ca(CH_3_COO)_2_·H_2_O synthesized from CaCO_3_ and three different acetic acid concentrations are shown in Fig. 3. The corresponding infrared adsorption characteristics (vibration mode and vibrational position (wavenumber)) are presented in Table 3. The vibrational characteristics of products were explained in detail. Two broad bands in the wavenumber range from 3687 to 3390 and 3380−3089 cm^−1^ are respectively assigned to the asymmetric *ν*_as_(O–H) and symmetric *ν*_s_(O–H) stretching vibrational modes of water (H_2_O). Two weak bands at 3041−2981 and 2955−2908 cm^−1^ ranges are respectively assigned to the asymmetric *ν*_as_(H_2_C–H) and symmetric *ν*_s_(H_2_C–H) stretching vibrational modes of methyl (CH_3_) group of acetate (CH_3_COO^−^) anion. These observations are in good agreement with the vibrational spectroscopic results reported by Musumeci et al. [54] for Ca(CH_3_COO)_2_·H_2_O/Ca(CH_3_COO)_2_·0.5H_2_O, Bette et al. [56] for Ca(CH_3_COO)_2_·H_2_O/(Ca(CH_3_COO)_2_) and Thongkam et al. [43] for Ca(CH_3_COO)_2_·H_2_O produced from scallop shells as a raw agent. A broad band from 1702 to 1567 cm^−1^ is assigned as both asymmetric *ν*_as_(C]O) and symmetric *ν*_s_(C]O) stretching vibrational modes of C]O bond of CH_3_COO^−^. A broadband from 1559 to 1505 cm^−1^ is assigned as the asymmetric *ν*_*a*s_(C–O) stretching vibrational mode, whereas a medium broad band observed at 1482–1431 cm^−1^ is assigned as the symmetric *ν*_s_(C–O) stretching vibration of C–O bond of CH_3_COO^−^. A medium broad band at 1425–1384 cm^−1^ is assigned as the asymmetric *δ*_as_(CH_3_) bending vibrational mode, whereas the small broad band presented at 1363–1328 cm^−1^ is assigned as symmetric *δ*_s_(CH_3_) bending vibrational mode of CH_3_ group of CH_3_COO^−^. The out-of-plane *ρ*_op_(CH_3_) stretching vibrational mode of CH_3_ of CH_3_COO^−^ was observed at the wavenumber region from 1080 to 1040 cm^−1^. Whereas the in-plane *ρ*_ip_(CH_3_) bending vibrational mode of CH_3_ of CH_3_COO^−^ appeared two broad peaks at the region from 1040 to 979 cm^−1^. The ν(C–C) stretching vibrational mode of C–C bond of CH_3_COO^−^ has split into three peaks at the region from 971 to 918 cm^−1^. Two peaks in the region from 694 to 651 cm^−1^ is assigned as the symmetric twisting *δ*_st_(O–C–O) and rocking *δ*_sr_(O–C–O) vibrational modes of O–C–O bonds of CH_3_COO^−^. A broadband from 651 to 592 cm^−1^ region is assigned as the out-of-plane *ρ*_op_(O–C–O) stretching vibrational mode of O–C–O bonds of CH_3_COO^−^.

**Table 3:** Summarized vibrational characteristics (mode) and vibrational positions (wavenumber/cm^−1^) observed in cockle-shell derived Ca(CH_3_COO)_2_·H_2_O products prepared from three different acetic acid concentrations.

Vibrational modes	Vibrational symbols	Wavenumber/cm^−1^
*Wavenumber region: 4000−2500 cm−1*		
O–H asymmetric stretching of H_2_O	*ν*_as_(O–H)	3687–3390
O–H symmetric stretching of H_2_O	*ν*_s_(O–H)	3380–3089
C–H asymmetric stretching of CH_3_ of CH_3_COO^−^	*ν*_as_(H_2_C–H)	3041–2981
C–H symmetric stretching of CH_3_ of CH_3_COO^−^	*ν*_s_(H_2_C–H)	2955–2908
*Wavenumber region: 2500−1200 cm−1*		
C]O asymmetric and symmetric stretching of CH_3_COO^−^	*ν*_as_(C]O) and *ν*_s_(C]O)	1702–1567
C–O asymmetric stretching of CH_3_COO^−^	*ν*_as_(C–O)	1559–1505
C–O symmetric stretching of CH_3_COO^−^	*ν*_s_(C–O)	1482–1431
C–H asymmetric bending of CH_3_	*δ*_as_(CH_3_)	1425–1384
C–H symmetric bending of CH_3_	*δ*_s_(CH_3_)	1363–1328
*Wavenumber region: 1200−400 cm^−1^*		
Out-of-plane bending of CH_3_	*ρ*_op_(CH_3_)	1080–1040
In-plane bending of CH_3_	*ρ*_ip_(CH_3_)	1040–979
C–C stretching vibration of CH_3_COO^−^	ν(C–C)	971–918
O–C–O symmetric twisting and rocking of CH_3_COO^−^	*δ*_st_(O–C–O) and *δ*_sr_(O–C–O)	694–651
O–C–O out-of-plane stretching of CH_3_COO^−^	*ρ*_op_(O–C–O)	651–592
Ca–O stretching	*ν*(Ca–O)	511–450

In addition, the stretching vibrational characteristic bands of metal−oxide bond *ν*(Ca–O) are observed at the wavenumber region from 511 to 450 cm^−1^. These spectroscopic results are like those reported by Musumeci et al. [54] Koleva [57], and May et al. [58]. All vibrational characteristics obtained in this experimental work confirmed the formation of cockle shell derived Ca(CH_3_COO)_2_·H_2_O compound. The vibrational characteristics of all synthesized Ca(CH_3_COO)_2_·H_2_O compounds are in good agreement to the results reported by Thongkam et al. [43], Seesanong et al. [45], Musumeci et al. [54] and Koleva [57].

### 3.6 Scanning electron microscope (SEM) results

**Fig. 4:**
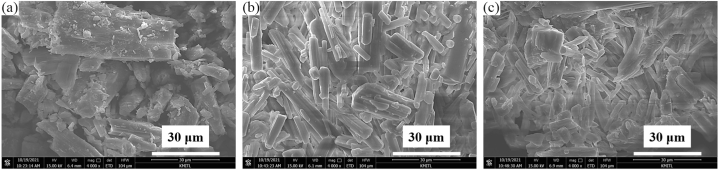
Scanning electron microscope (SEM) images of the synthesized calcium acetate monohydrate products ((a) CAM8, (b) CAM10, and (c) CAM12) prepared by using different acetic acid concentrations.

The morphologies of all cockle-shell derived Ca(CH_3_COO)_2_·H_2_O products are demonstrated in Fig. 4. The SEM images of Ca(CH_3_COO)_2_·H_2_O ((a) CAM8, (b) CAM10, and (c) CAM12) samples synthesized from three different acetic acid concentrations (8, 10, and 12 mol L^−1^) were analyzed to investigate the influence of the concentration of acetic acid on the morphology. As demonstrated in Fig. 4 (a), using the lowest acetic acid concentration (8 mol L^−1^), the morphology result of CAM8 shows the coalescence of large plate-shaped crystals with different sizes from around 5−50 μm. On the other hand, when higher concentrations of acetic acid (10 and 12 mol L^−1^) were used in the calcium acetate synthesis, the morphology results of CAM10 (Fig. 4 (b) and CAM12 (Fig. 4(c)) samples show many rod-shaped crystals in the range of 4–15 μm and 2−10 μm, respectively. However, some plate-shaped crystals were also observed for both CAM10 (Fig. 4 (b) and CAM12 (Fig. 4(c)) samples. According to the SEM results, it might be assumed that the concentration of acetic acid used in the synthesis process acted as an important influencer on the morphology of products. Increasing acetic acid concentration can reduce the particle size of the synthesized Ca(CH_3_COO)_2_·H_2_O crystals. The morphology (size and shape) of all synthesized Ca(CH_3_COO)_2_·H_2_O in this work are significantly different from an agglomeration like-timbers with dimensions in the range of 2–60 μm of Ca(CH_3_COO)_2_·H_2_O produced from scallop shell (a raw agent) in our previous report [43].

### 3.7 Thermogravimetric analysis (TGA) results

**Fig. 5:**
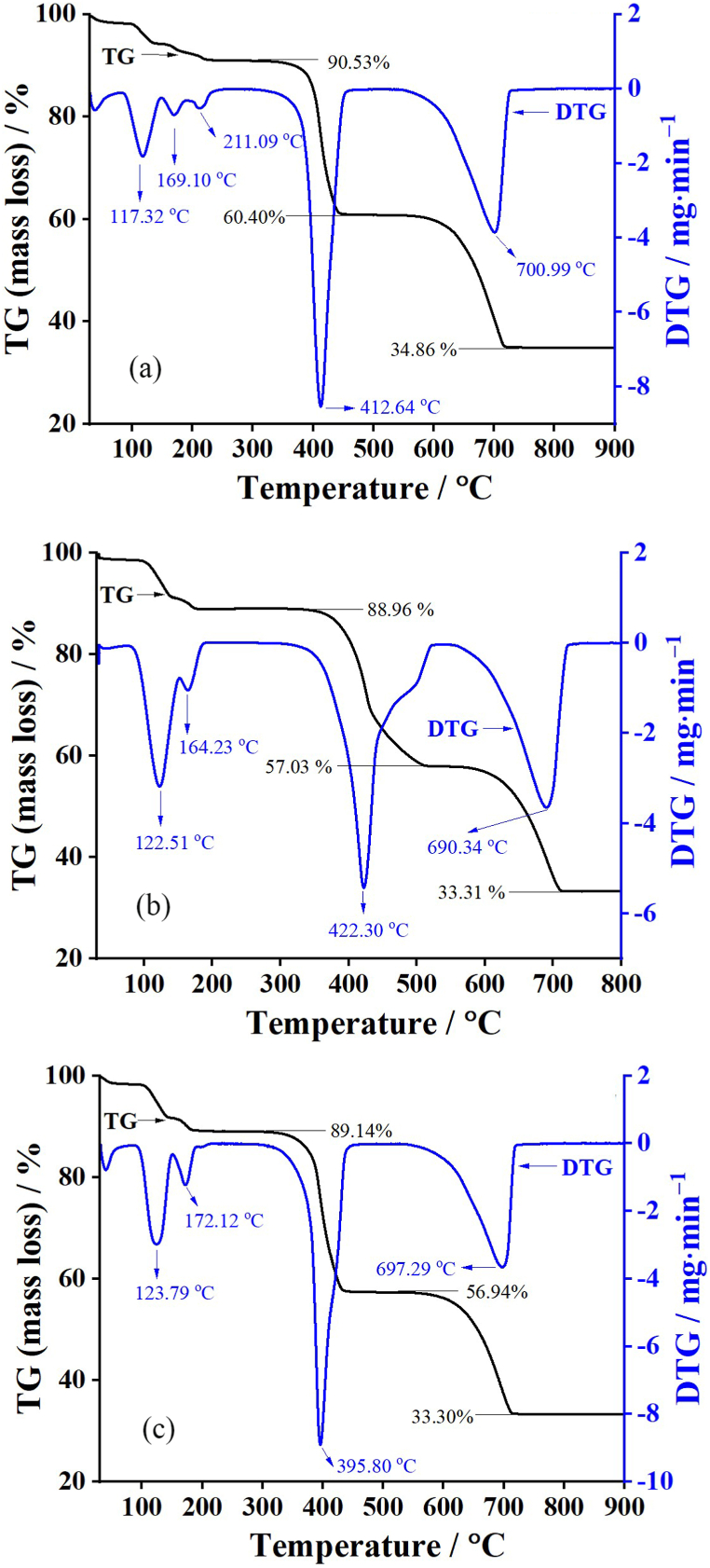
Thermal decomposition (TG and DTG) curves of the synthesized calcium acetate monohydrate products ((a) CAM8, (b) CAM10, and (c) CAM12) prepared from three different acetic acid concentrations.

Fig. 5 shows the TG and DTG curves of all Ca(CH_3_COO)_2_·H_2_O samples ((a) CAM8, (b) CAM10, and (c) CAM12) in the temperature range of 30–900 °C. According to the results, different acetic acid concentrations slightly affect the thermal decomposition curves of Ca(CH_3_COO)_2_·H_2_O compounds.

The thermal decomposition processes of samples were described. The first mass-loss step, observed at the temperature range between 30 and 300 °C, corresponded to the evaporation of water (dehydration process) from Ca(CH_3_COO)_2_·H_2_O. Eq. (9) showed the dehydration reaction of Ca(CH_3_COO)_2_·H_2_O and the thermal decomposition product was formed as anhydrous Ca(CH_3_COO)_2_. The corresponding DTG curves are shown as triple peaks for CAM8 (117, 169, and 211 °C) (Fig. 5 (a)) and double peaks for CAM10 (123 and 172 °C) (Fig. 5 (b)) and CAM12 (123 and 164 °C) (Fig. 5 (c)). This result is the effect of inter- and intramolecular interaction of water molecules in each compound with the different surroundings.

(9) First decomposition step: Dehydration process Ca(CH_3_COO)_2_·H_2_O(s) → Ca(CH_3_COO)_2_(g) + H_2_O(g)

This thermal decomposition product (Ca(CH_3_COO)_2_) agrees with the result reported by Bette et al. [59]. When Ca(CH_3_COO)_2_·H_2_O was calcined at the temperature of 300 °C, anhydrous Ca(CH_3_COO)_2_ was obtained. The determined mass loss observed from the samples with a mass loss of about 10 % agreed with the theoretical value (10.23 %), corresponding to the water evaporation. This dehydration process can be interpreted as the dehydration of both water sites: outer and inner crystal structures. The outer water was assigned as the moisture or water that was adsorbed on the crystal surface of Ca(CH_3_COO)_2_·H_2_O, and this adsorbed water was called “physisorbed water”. In contrast, the inner water was the water that grew in the crystal structure of Ca(CH_3_COO)_2_·H_2_O.

As the temperature increased, the second thermal decomposition process was obtained at temperatures between 300 and 510 °C and related to the mass loss of about 32 %. This thermal process is assigned as the decomposition of anhydrous Ca(CH_3_COO)_2_ and the CaCO_3_ and acetone (CH_3_COCH_3_) compounds were then thermal generated [59]. At the same time, the generated CH_3_COCH_3_ further decomposed immediately to ketene (H_2_CCO) and methane (CH_4_) [60]. Eq. (10) showed the decomposition reaction of anhydrous Ca(CH_3_COO)_2_ and the thermal decomposition product was formed as CaCO_3_. This thermal decomposition step was call “deacetonation process”. The respective DTG peaks are observed close to the same at 412, 395, and 422 °C for CAM8 (Fig. 5 (a)), CAM10 (Fig. 5 (b)), and CAM12(Fig. 5 (c)), respectively.

(10) Second decomposition step: Deacetonation process Ca(CH_3_COO)_2_ (s) → CaCO_3_(s) + CH_3_COCH_3_(g)

The final thermal decomposition step was obtained at temperatures between 510 and 740 °C and related to the mass loss of about 23 %. This process is assigned as the thermal decomposition of CaCO_3_ and then generates solid-state CaO and CO_2_ gas [59] as shown in Eq. (11). The relative DTG peaks are observed close to the same at 700, 697, and 690 °C for CAM8 (Fig. 5 (a)), CAM10 (Fig. 5 (b)), and CAM12(Fig. 5 (c)), respectively.

(11) Final decomposition step: Decarbonization process CaCO_3_(s) → CaO(s) + CO_2_(g)

The total mass loss and the mass retained were about 67 % and 33 % (>690 °C), respectively, forming CaO as a final stable substance. The total mass loss and the residual mass of all prepared Ca(CH_3_COO)_2_·H_2_O products were well consistent with theoretical data as 68 % and 32 % according to reactions, respectively. However, this result may not be in agreement with Ca(CH_3_COO)_2_·H_2_O obtained from oyster shells (>800 °C) [31] and scallop shells (>700 °C) [43] reported a higher temperature to get the CaO.

Based on these observed results, the calcium acetate product prepared from the cockle shell shows differences in thermal stability, morphological structure purity, and metal contamination from those reported obtained from other sources and shell types in the previous works [31,43]. This research is therefore extremely useful for communities that have cockle shell sources as the only raw material in calcium acetate production which may be used on an industrial production scale.

## 4 Conclusions

Usage of the waste for the production of other compounds can decrease the environmental problem and increase the value of the used waste. In this research, cockle shell waste was successfully employed as a renewable source to synthesize calcium acetate. The effect of different acetic acid concentrations (8, 10, and 12 mol L^−1^) on the reaction temperature, drying time, % yield, and the physicochemical properties of calcium acetate products were also investigated. XRF and XRD results confirmed the chemical composition and crystallography of the synthesized products. The plate-shaped and rod-shaped crystals of products were observed from the SEM images, which demonstrated that the sample particle sizes decreased with increasing acetic acid concentration. Thermal analysis exhibited three major thermal decomposition processes: *i* dehydration, *ii* deacetonation, and *iii* decarbonization processes, respectively, and its final thermal decomposed product as CaO. All experimental results confirmed that all synthesized products from the reaction between cockle shell waste powder and acetic acid were Ca(CH_3_COO)_2_·H_2_O. In addition, Ca(CH_3_COO)_2_·H_2_O synthesized from 10 mol L^−1^ acetic acid (CAM10) showed the highest % yield (96.30 %), maximum calcium content (96.2 % CaO) with lower other impurities and short time consumption of 17 h. The work presents calcium acetate production from cockle shells with a rapid and easy process that leads to differences in thermal stability, morphological structure purity, %yield, and metal contamination of the obtained product with those reported obtained from other sources and shell types in the previous work and resulted in cheap obtained chemical compound costs. The obtained results pointed out that the transformation of cockle shells to calcium carbonate, replacing calcium ores used for calcium acetate production, can help to reduce the usage of calcium mineral resources (limited and non-renewable sources) and greenhouse gas emissions, which offers a promising pathway for effectively managing sustainable environments at an industrial scale.

## Additional information

No additional information is available for this paper.

## Data availability statement

All data are fully available without restriction.

## Compliance with ethics requirements

This article does not contain any studies with human or animal subjects.

## Funding

This work is a result of the project entitled “Conversion of shell/eggshell biowastes for sustainable environmental remediation “Grant No. RE-KRIS/FF67/030 by King Mongkut's Institute of Technology Ladkrabang (10.13039/501100007120KMITL), which has received funding support from the NSRE.

## CRediT authorship contribution statement

**Somkiat Seesanong:** Writing – review & editing, Writing – original draft, Conceptualization. **Chaowared Seangarun:** Investigation, Formal analysis, Data curation. **Banjong Boonchom:** Writing – review & editing, Visualization, Supervision, Resources, Project administration, Methodology, Funding acquisition, Conceptualization. **Natee Ohpasee:** Investigation. **Nongnuch Laohavisuti:** Visualization, Supervision, Project administration, Funding acquisition, Data curation, Conceptualization. **Wimonmat Boonmee:** Writing – review & editing, Writing – original draft, Visualization, Data curation. **Pesak Rungrojchaipon:** Methodology, Investigation, Funding acquisition.

## Declaration of competing interest

The authors declare that they have no known competing financial interests or personal relationships that could have appeared to influence the work reported in this paper.
