# Supplementary material for: Green Ca-source of cockle shells converted to calcium acetate for environmental sustainability
Source: Heliyon. 2024 May 31;10(11):e32153. doi: 10.1016/j.heliyon.2024.e32153 (PMC11168426; doi:10.1016/j.heliyon.2024.e32153)
Supplement: Multimedia component 1 [file mmc1.docx]

1. Calculating CO_2_ emissions from the production of the bio-green CaCO_3_ powder obtained from cockle shell wastes is based on the following equation [1, 2]:

E _bio-green CaCO3_ = E_T_ + E_W_ + E_E_ (S1)

E_T_ = distance x loading x EF_tuck_ (S2)

E_W_ = amount of water usage x EF_water_ (S3)

E_E_ = electricity consumption x EF_E_ (S4)

E _bio-green CaCO3_  is the carbon emission of the production of the bio-green CaCO_3_ powder, kgCO_2_

E_T_ is the carbon emission of transportation of the production of the bio-green CaCO_3_ powder, kgCO_2_

E_W_ is the carbon emission of water usage for the production of the bio-green CaCO_3_ powder, kgCO_2_

E_E_ is the carbon emission of electricity consumption for the production of the bio-green CaCO_3_ powder, kgCO_2_

EF_tuck_ is an emission factor for a kind of tuck; EF_W_ is an emission factor for water; EF_E_ is an emission factor for electricity

The calculated data of CO_2_ emissions in the production of the bio-green CaCO_3_ powder obtained from cockle shell wastes based on data from Fig. S1 using Eq. (S1) are tabulated in Table S1

1. Calculating CO_2_ emissions from the production of calcium acetate from the reaction of the bio-green CaCO_3_ powder obtained from cockle shell wastes and various acetic acid concentrations in the following reaction (S5) and equation (S6) [3, 4]:

CaCO_3_(s) + 2CH_3_COOH(aq) ⟶ Ca(CH_3_COO)_2_·H_2_O(s) + CO_2_(g) (S5)

E_Ca(CH3COO)2·H2O_ = E_CaCO3_ + E_CH3COOH_ + E_CO2_ (S6)

E_CaCO3_ is the carbon emission of a number of CaCO_3_(s) usage x EF _CaCO3_  E_CH3COOH_ is the carbon emission of a number of 2CH_3_COOH(aq) x EF_CH3COOH_  E_CO2_ is the carbon emission of the formation of CO_2_ in the chemical reaction (5) = 44/100 kgCO_2_

The calculated results of CO_2_ emissions in the production of calcium acetate from reaction (S5) by Eq (S6) are shown in Table S2.

**Table S1** Calculation of carbon emissions in the production of the bio-green CaCO_3_ powder obtained from cockle shell wastes [1-3].

| **Activity data** | **Details** | **Quantity** | **unit** | **EF** | **Carbon emissions (kgCO_2_e/kg)** |
| --- | --- | --- | --- | --- | --- |
| Transportation | 10-wheel (16-ton truck/time) | 75 | km | 0.0649 kgCO_2_e/ tkm | 0.0049 |
| Chemical usage | NaOCl 15 %Cl_2_ | 0.17 | L | 0.7812 kgCO2e/L | 0.1328 |
|  | NaOCl 2.5 %Cl_2_  (1 kg cleaned Shells) | 1 | L | 0.1345 kgCO2e/L | 0.1345 |
| water | DI water | 0.83 | L | 0.0020 kgCO2e/L | 0.0017 |
| Electricity | Electricity (100 kg/h) | 1.5 | kwh | 0.5986 kgCO_2_e/kwh | 0.0090 |
| **Carbon emissions in the production of the bio-green CaCO_3_ powder** | | | | | **0.1492** |

Remarks: The sunlight process or drying is no carbon emissions.

**Table S2** Calculation of carbon emissions in the production of calcium acetate from the reaction of the bio-green CaCO_3_ powder obtained from cockle shell wastes and various acetic acid concentrations[1-4].

| **Chemical agent** | **Details** | **Quantity** | **unit** | **EF** | **Carbon emissions**  **(kgCO_2_e/kg)** |
| --- | --- | --- | --- | --- | --- |
| CaCO_3_(s) | Bio-green CaCO_3_ from cockle shells (94.7% purity) | 1.06 | kg | **0.1492** kgCO_2_e/ kg | 0.1582 |
|  | Minerals(Lime or rock stone) | 1.00 | kg | 1.067 kgCO_2_e/ kg | 1.0670 |
| CH_3_COOH | 17.416 mol·L^−1^ | 1.00 | L | 2.6987 kgCO_2_e/L | - |
|  | 12 mol·L^−1^ | 1.67 | L | 1.8601 kgCO_2_e/L | 3.1064 |
|  | 10 mol·L^−1^ | 2.00 | L | 1.5504 kgCO_2_e/L | 3.1008 |
|  | 8 mol·L^−1^ | 2.50 | L | 1.2407 kgCO_2_e/L | 3.1018 |
| Ca(CH_3_COO)_2_·H_2_O  (Mw =176 g/mol)  obtained from Bio-green CaCO_3_ | CA12 (94.90% yield ) | 1.67 | kg | - | (0.1582+3.1064+0.440)/1.67 = 2.2183 |
|  | CA10 (96..30% yield ) | 1.69 | kg | - | (0.1582+3.1008+0.440)/1.69 = 2.1888 |
|  | CA8 (91.88% yield ) | 1.62 | kg | - | (0.1582+3.1018+0.440)/1.62 = 2.2840 |
| Ca(CH_3_COO)_2_·H_2_O  Obtained from minerals react with acetic acids | 12 mol·L^−1^  (estimated 100 % yield ) | 1.76 | kg | - | (1.067 +3.1064+0.440)/1.76 = 2.6213 |
|  | 10 mol·L^−1^  (estimated 100 % yield ) | 1.76 | kg | - | (1.067 +3.1008+0.440)/1.76 = 2.6181 |
|  | 8 mol·L^−1^  (estimated 100 % yield ) | 1.76 | kg | - | (1.067+3.1018+0.440)/1.76 = 2.6186 |

**Remarks:** The carbon emissions of various acetic acid concentrations estimated from the dilution of 17.416 mol·L^−1^acetics acid by DI water (EF_DI_ = 0.0020 kgCO_2_e/L)

Transportation

75 km

(Ladkrabang, BKK)

Cockle shell wastes

(Bang Saen beaches, Chonburi province Thailand)

Bio-green CaCO_3_

powders

Grinding and Sieve

(electricity)

Drying

(Sunlight)

Cleaning

(NaOCl)

**Figure S1** Process line of production for the bio-green CaCO_3_ powder obtained from cockle shell wastes

**References**

1. NSTDA-TIIS, Establishment of Thai National Life Cycle Inventory Database, Bangkok: National Science and Technology Development Agency-Technology and Informatics Institute for Sustainability; 2012 [in Thai]. https://www.nstda-tiis.or.th/en/publications_media/thai-lci-database-manual-2/ (Accessed 23 Jul 2023)
2. TGO, Guidelines for assessing the carbon footprint of products, Bangkok: Thailand Greenhouse Gas Management Organization (Public Organization); 2018 [Thai]. <http://thaicarbonlabel.tgo.or.th/index> (Accessed 23 Jul 2023).
3. EIPPCB, Large Volume Inorganic Chemicals – Solids and Others Industry, Seville: European Integrated Pollution Prevention and Control Bureau; 2007. <https://eippcb.jrc.ec.europa.eu/sites/default/files/2019-11/lvic-s_bref_0907.pdf> (Accessed 23 Jul 2023).
4. IPCC, 2006 IPCC Guidelines for National Greenhouse Gas Inventories, Geneva: Intergovernmental Panel on Climate Change; 2006. <https://www.ipcc-nggip.iges.or.jp/public/2006gl/vol3.html> (Accessed 23 Jul 2023).
